# The In Vitro Anti-Tumor Activity of Phycocyanin against Non-Small Cell Lung Cancer Cells

**DOI:** 10.3390/md16060178

**Published:** 2018-05-23

**Authors:** Shuai Hao, Yan Yan, Shuang Li, Lei Zhao, Chan Zhang, Liyun Liu, Chengtao Wang

**Affiliations:** 1 Beijing Advanced Innovation Center for Food Nutrition and Human Health, Beijing Engineering and Technology Research Center of Food Additives, Beijing Technology and Business University, Beijing 100048, China; shmilyhs321@163.com (S.H.); 15128470659@163.com (Y.Y.); lishuangldw@163.com (S.L.); zhaolei@th.btbu.edu.cn (L.Z.); zhangchan@th.btbu.edu.cn (C.Z.); 2 State Key Laboratory of Infectious Disease Prevention and Control, National Institute for Communicable Disease Control and Prevention, Collaborative Innovation Center for Diagnosis and Treatment of Infectious Disease, Chinese Center for Disease Control and Prevention, Beijing 102206, China

**Keywords:** phycocyanin, non-small cell lung cancer, proliferation, apoptosis, NF-κB signaling

## Abstract

Phycocyanin, a type of functional food colorant, is shown to have a potent anti-cancer property. Non-small cell lung cancer (NSCLC) is one of the most aggressive form of cancers with few effective therapeutic options. Previous studies have demonstrated that phycocyanin exerts a growth inhibitory effect on NSCLC A549 cells. However, its biological function and underlying regulatory mechanism on other cells still remain unknown. Here, we investigated the in vitro function of phycocyanin on three typical NSCLC cell lines, NCI-H1299, NCI-H460, and LTEP-A2, for the first time. The results showed that phycocyanin could significantly induce apoptosis, cell cycle arrest, as well as suppress cell migration, proliferation, and the colony formation ability of NSCLC cells through regulating multiple key genes. Strikingly, phycocyanin was discovered to affect the cell phenotype through regulating the NF-κB signaling of NSCLC cells. Our findings demonstrated the anti-neoplastic function of phycocyanin and provided valuable information for the regulation of phycocyanin in NSCLC cells.

## 1. Introduction

Extensive research has suggested that many natural products derived from food and food supplements have various health-promoting effects [1]. Recently, marine natural products with pharmacological activity have been shown to have multiple potent biological functions, with less or no toxic side effects [2]. Thus, they have become one of the most important resources of novel lead compounds for critical diseases, which have seen important development and utilization in recent years [3]. Phycocyanin, a marine natural blue photosynthetic pigment protein purified from *Spirulina*, is one of the accepted natural functional foods around the world [4]. Phycocyanin has excellent anti-tumor activity. Studies have shown that phycocyanin plays anti-proliferation and pro-apoptotic effects on different cancer cell lines in vitro, while it has no side effects on normal tissue cells [5,6]. In addition, it also shows good therapeutic values such as antioxidant, anti-inflammatory, immunomodulation, blood vessel-relaxing, and blood liquid-lowering activities, etc. [7,8,9,10,11]. Thus, further investigating on the function and mechanism of phycocyanin has important guiding significance and research value.

Several studies have demonstrated that phycocyanin has bioactivity in kinds of cancers, including breast cancer [6,12], histiocytic tumor [13], ovarian cancer [14], colon cancer [15], prostate cancer [16], melanoma, and lung cancer [17]. Among them, lung cancer is one of the most common health threats in the world, especially given its high mortality rates [18,19]. Human lung cancer is generally classified into two major categories, small cell lung cancer (SCLC) and non-small cell lung cancer (NSCLC). NSCLC is responsible for more than 85% of all lung carcinoma cases, with the characteristics of higher mortality, lower cure rate, and stronger metastasis [20]. Therefore, exploring the anti-cancer function and underlying mechanism of phycocyanin on NSCLC is critical.

The inhibiting effect of phycocyanin on NSCLC has been reported in several studies. Li et al. revealed that phycocyanin could inhibit the growth of NSCLC A549 cells in vivo and in vitro, which also has a synergistic anti-tumor effect with all-trans retinoic acid [21,22]. Bingula et al. reported that phycocyanin and betaine have a synergistic inhibiting effect on the viability of A549 cells [23]. Baudelet et al. discovered that glaucophyte *Cyanophora paradoxa* extracts could significantly inhibit the growth of three cancer cell lines, including A549 cells [17]. However, the abovementioned studies all investigated the function of phycocyanin in one single NSCLC cell line. Moreover, the anti-lung cancer mechanism of phycocyanin remains unclear. Herein, we investigated the growth inhibitory effects and underlying mechanism of phycocyanin in three human NSCLC cell lines, NCI-H1299, LTEP-A2, and NCI-H460. The results laid a solid theoretical foundation for the treatment of NSCLC and the development and utilization of phycocyanin.

## 2. Results

### 2.1. Phycocyanin Induces Morphological Changes in NSCLC Cells

**Figure 1:**
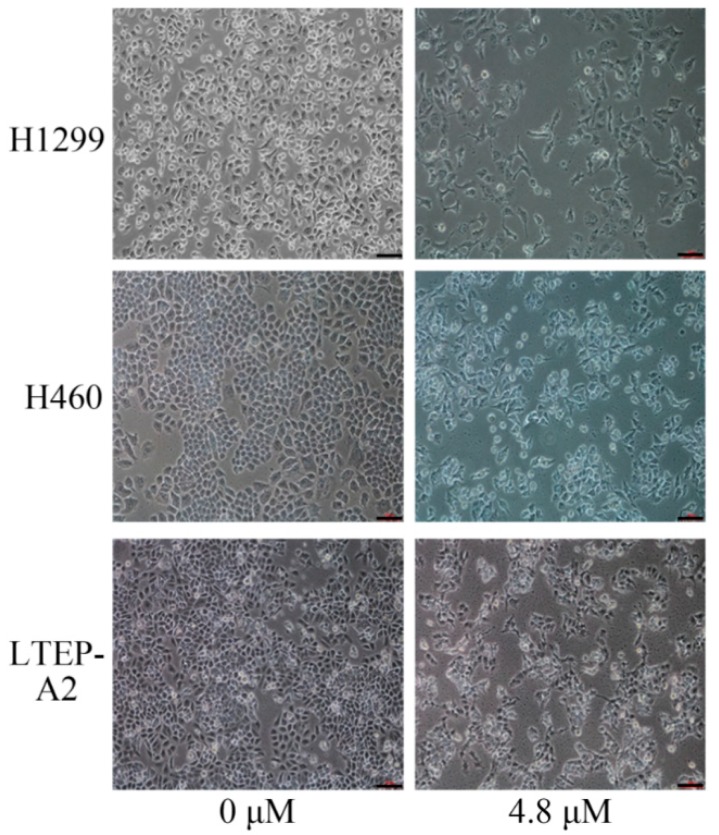
Phycocyanin induces morphological changes in non-small cell lung cancer (NSCLC) cells. H1299, H460, and LTEp-A2 cells were treated with different doses (0 and 4.8 μM) of phycocyanin for 72 h, and photographed under a light microscope (100×). Scale bars represent 100 μm.

To address the relationship between phycocyanin and its effect on non-small cell lung cancer, the morphology of NSCLC cells, H1299, H460, and LTEP-A2, was first studied upon treatment with various doses of phycocyanin. As shown in Figure 1, the normal morphology of H1299 cells was fusiform or triangular. After treatment with 4.8 μM phycocyanin for 72 h, cells appeared in anomalous forms, some of which became needle-shaped. Similarly, the morphology of H460 and LTEP-A2 cells were also abnormally changed by phycocyanin. Furthermore, the number of cells was obviously reduced after phycocyanin treatment. These results suggested that phycocyanin might have a pro-apoptotic effect on NSCLC cells.

### 2.2. Phycocyanin Induces Apoptosis in NSCLC Cells

**Figure 2:**
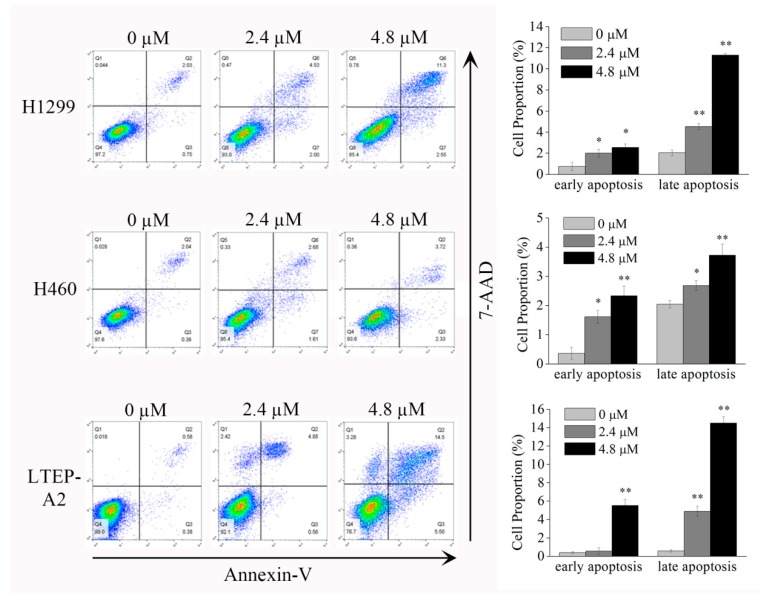
Phycocyanin induces apoptosis in NSCLC cells. H1299, H460, and LTEP-A2 cells were incubated with different concentrations of phycocyanin (0, 2.4, and 4.8 µM) for 48 h and subjected to apoptosis tests. The proportion of early apoptotic and late apoptotic cells were analyzed. Bars represent mean ± SD. *, *p* < 0.05; **, *p* < 0.01.

As phycocyanin induces morphological changes in NSCLC cells, we next studied the extent of apoptosis in H1299, H460, and LTEP-A2 cells by Annexin V-FITC and 7AAD staining. Figure 2 shows that phycocyanin-treated NSCLC cells demonstrated an induction of apoptosis in comparison to untreated cells. The late apoptotic percentages of H1299 (4.53 ± 0.27%), H460 (2.68 ± 0.37%), and LTEP-A2 cells (4.88 ± 0.55%) increased after incubation with 2.4 µM phycocyanin, as compared to the control groups. In addition, the apoptosis degree of NSCLC cells presented a dose-dependent effect with phycocyanin. A high concentration of phycocyanin (4.8 µM) significantly increased the late apoptotic percentages of H1299 (11.30 ± 0.16%), H460 (3.72 ± 0.98%), and LTEP-A2 cells (14.50 ± 0.68%).

**Figure 3:**
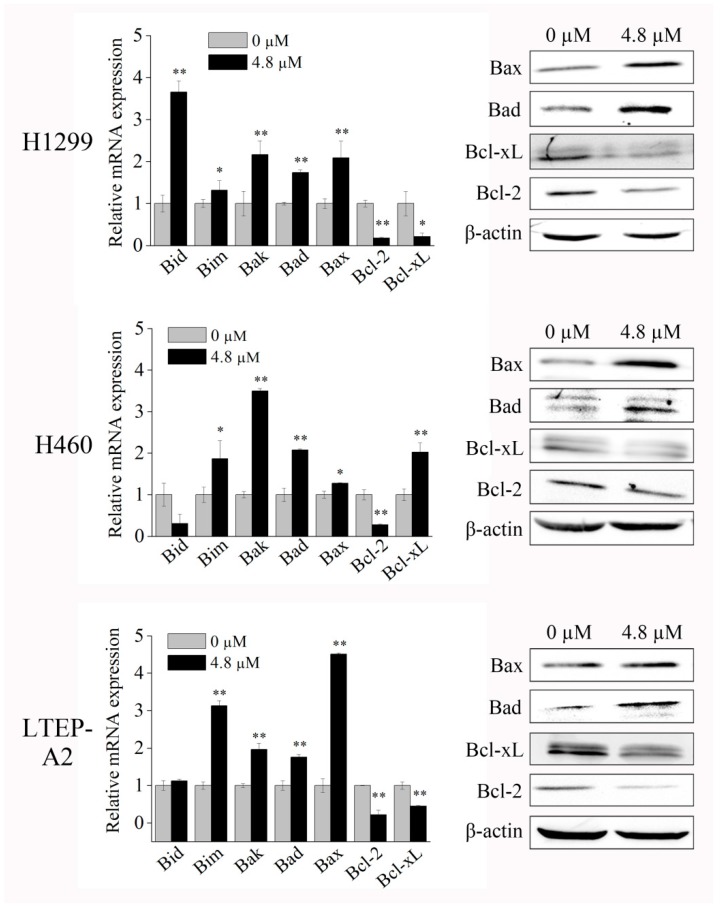
Quantitative real-time PCR (qRT-PCR) and Western blot analysis of apoptotic markers in NSCLC cells after phycocyanin treatment. H1299, H460, and LTEP-A2 cells were incubated with 4.8 µM phycocyanin. qRT-PCR and Western blot were performed at 24 h and 48 h after treatment, respectively. Bars represent mean ± SD. *, *p* < 0.05; **, *p* < 0.01.

To gain a deeper insight into the mechanism of apoptosis induced by phycocyanin, we tested the expressions of apoptotic markers using quantitative real-time PCR (qRT-PCR) and Western blot. As shown in Figure 3, phycocyanin could significantly increase the transcriptional levels of pro-apoptotic genes *Bim*, *Bak*, *Bax,* and *Bad*, in addition to reducing the levels of *Bcl-xL* and *Bcl-2*, two anti-apoptotic genes in H1299 and LTEP-A2 cell lines. These results were further supported by Western blot analysis. It was interesting to find that in H460 cells, although the protein level of Bcl-xL was downregulated, its transcriptional level increased after phycocyanin treatment. In addition, the protein level of Bcl-2 showed no obvious alteration while its transcriptional level significantly decreased. These results indicated that a post-transcriptional mechanism might involve the regulation of these genes in H460 cells. Taken together, the above results suggested that phycocyanin could induce apoptosis in NSCLC cells.

### 2.3. Phycocyanin Displays Anti-Migratory Effect against NSCLC Cells

**Figure 4:**
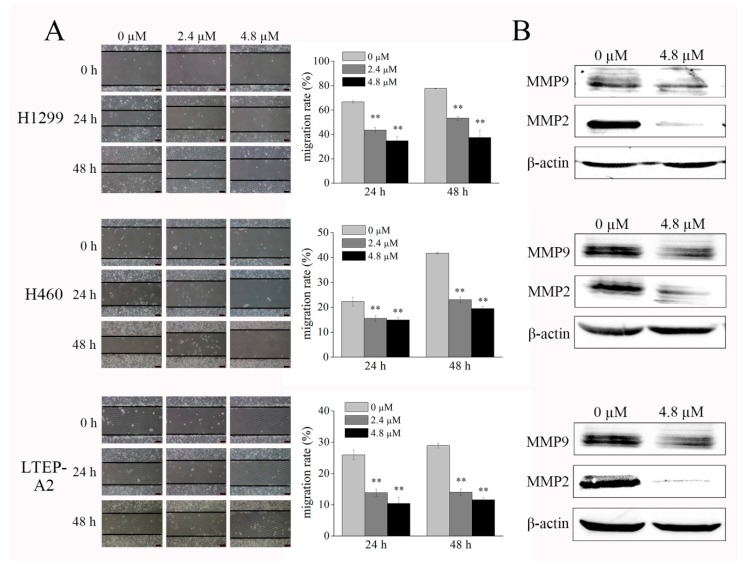
Phycocyanin displays anti-migratory effect against NSCLC cells. (**A**) The wound-healing assay showed representative effects of phycocyanin (0, 2.4, and 4.8 µM) on H1299, H460, and LTEP-A2 cell migration at 24 and 48 h. Quantification of wound closure was shown by histogram. Scale bars represent 100 µm. (**B**) Western blot analysis of MMP2 and MMP9 expression in NSCLC cells after 4.8 µM phycocyanin treatment for 48 h. MMP2, matrix metalloproteinase-2; MMP9, matrix metalloproteinase-9. Bars represent mean ± SD. **, *p* < 0.01.

A wound-healing assay was employed to determine the effect of phycocyanin on cell migration. As shown in Figure 4A, phycocyanin significantly suppressed the migration of H1299, H460, and LTEP-A2 cells in dose- and time-dependent manners (left panel); the migration rates were calculated and are presented in the right panel. After phycocyanin treatment (4.8 µM) for 48 h, the wound closure of H1299 cells clearly decreased from 77.60 ± 0.24% to 37.35 ± 6.24%. Similar results were found in H460 and LTEP-A2 cells. It is worth mentioning that in this study, we cultured cells with medium containing 3% instead of 10% fetal bovine serum (FBS), which eliminated the contribution of proliferation to the phycocyanin-induced inhibition of cell migration. Matrix metalloproteinase-2 (MMP2) and matrix metalloproteinase-9 (MMP9) are gelatinases of the matrix metalloproteinase family, which play a crucial role in cancer cell growth and migration due to their ability to degrade extracellular matrix proteins [24]. In present study, we found that phycocyanin treatment significantly reduced the expression of MMP2 and MMP9 in NSCLC cells (Figure 4B), which was in accordance with the wound-healing analysis. Taken together, these results suggested that phycocyanin displayed inhibitory activity on NSCLC cell migration. 

### 2.4. Phycocyanin Inhibits Proliferation and Colony Formation Ability of NSCLC Cells

**Figure 5:**
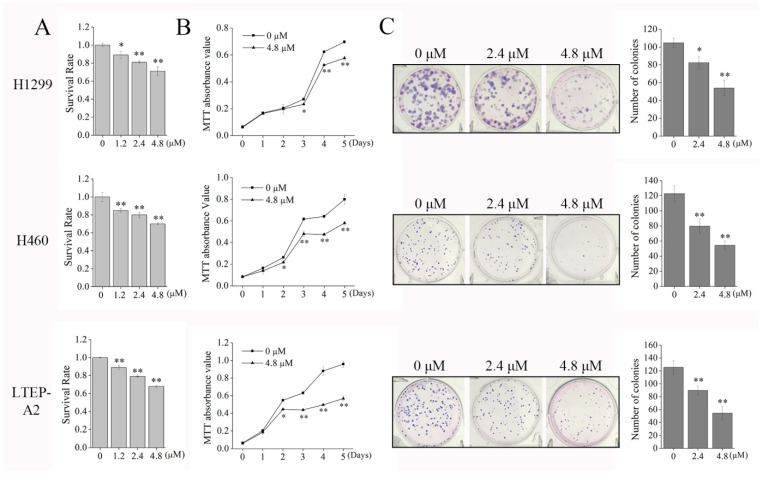
Phycocyanin inhibits the proliferation and colony formation ability of NSCLC cells. (**A**) Cell viability analysis of H1299, H460, and LTEP-A2 after different concentrations (0, 1.2, 2.4, and 4.8 μM) of phycocyanin treatment for 72 h. (**B**) MTT analysis of cell proliferation of H1299, H460, and LTEP-A2 after 4.8 μM phycocyanin treatment. (**C**) Colony formation assay of H1299, H460, and LTEP-A2 cells after treatment of different concentrations (0, 2.4, and 4.8 μM) of phycocyanin for 10–14 days. The quantitative representation of the reduction in number of colonies was shown by histogram. Bars represent mean ± SD. *, *p* < 0.05; **, *p* < 0.01.

The inhibitory effects of phycocyanin on the viability and proliferation of NSCLC cells were determined. As shown in Figure 5A, compared with control cells, incubation with phycocyanin (1.2, 2.4, and 4.8 µM) dose-dependently inhibited the viability of the three NSCLC cell lines. In addition, MTT assay (Figure 5B) showed that phycocyanin (4.8 µM) could significantly suppress the proliferation of NSCLC cells from the second (H460 and LTEP-A2 cells) or the third day (H1299 cells). These results suggested that phycocyanin exerted anti-proliferation effects on tested cells. To further establish the inhibitory role of phycocyanin on the transforming properties of NSCLC cells, we performed a clonogenic assay. Figure 5C shows that the three types of NSCLC cells could all form well-defined and distinct colonies, only the colonies of H1299 cells were bigger than the other two cell lines. Results showed that phycocyanin-treated cells displayed significant reduction in colony formation when compared to controls, which was indicative of potent inhibition of cell growth and reproductive integrity.

### 2.5. Phycocyanin Induces Cell Cycle Arrest in NSCLC Cells

**Figure 6:**
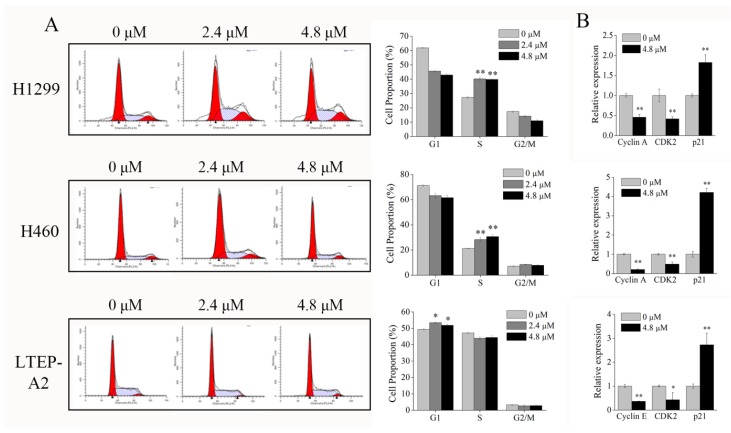
Phycocyanin induces cell cycle arrest in NSCLC cells. (**A**) Cell cycle analysis of NSCLC cells after treated with different concentrations of phycocyanin (0, 2.4, and 4.8 μM) for 48 h. (**B**) qRT-PCR analysis of the transcriptional levels of cell cycle regulatory genes at 24 h after phycocyanin treatment. Glyceraldehyde-3-phosphate dehydrogenase (GAPDH) was used as an internal control for normalization. Bars represent mean ± SD. *, *p* < 0.05; **, *p* < 0.01.

To elucidate the mechanism of growth inhibition on NSCLC cells, the effects of phycocyanin on cell cycle progression were determined in H1299, H460, and LTEP-A2 cells. Figure 6A showed that phycocyanin caused significant changes in the cell cycle distribution of H1299 and H460 cells. After incubation with 4.8 μM phycocyanin, the proportion of S phase cells reached 40.10 ± 1.06% and 30.60 ± 1.55% in H1299 and H460, respectively, as compared to the control groups (27.20 ± 0.80% and 21.10 ± 0.26% in H1299 and H460 cells, respectively), suggesting that phycocyanin caused S phase arrest in these two cell lines. Interestingly, unlike H1299 and H460 cells, phycocyanin (4.8 μM) induced a significant G1 phase increase (51.79 ± 0.80%) in LTEP-A2 cells compared to the control (49.39 ± 0.38%). Although phycocyanin could suppress the proliferation of NSCLC cells, the present results indicated that the inhibitory mechanism in LTEP-A2 might differ from that in H1299 and H460 cells. To further confirm the above results, we tested the levels of cell cycle regulatory genes involved in S/G2 and G1/S transition. As shown in Figure 6B, after phycocyanin treatment, Cyclin A and CDK2, two key positive regulators in S/G2 checkpoint [25], were significantly downregulated, whereas p21, an important cell cycle suppressor gene [26], was upregulated in H1299 and H460 cells. However, in LTEP-A2 cells, phycocyanin inhibited the transcriptional level of Cyclin E, a G1/S checkpoint regulator [27]. The qRT-PCR results strongly indicated that phycocyanin could trigger G1 phase arrest of LTEP-A2 cells and S phase arrest of H1299 and H460 NSCLC cells. 

### 2.6. Phycocyanin Reduces NF-κB Signaling Activity in NSCLC Cells

**Figure 7:**
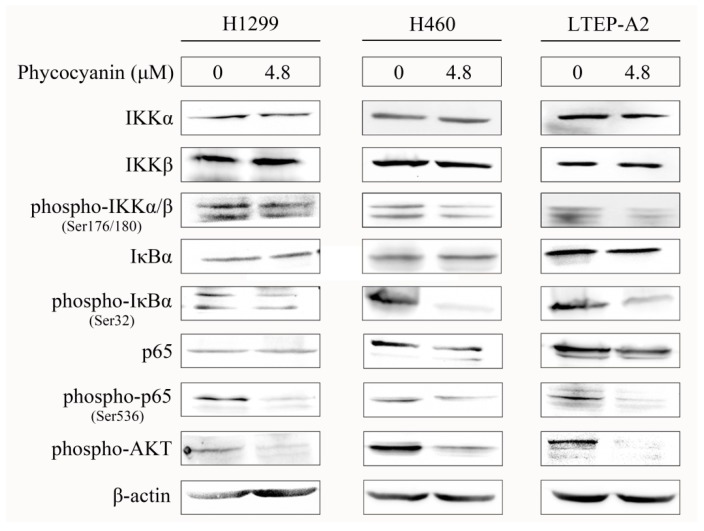
Phycocyanin reduces NF-κB signaling activity in NSCLC cells. The protein expressions of NF-κB signaling and phospho-AKT were analyzed by Western blot in H1299, H460, and LTEP-A2 cells after 0 and 4.8 μM phycocyanin treatment for 48 h.

To investigate the underlying regulatory mechanism of phycocyanin in NSCLC cells, we determined the protein expressions of NF-κB signaling in H1299, H460, and LTEP-A2 cells after 4.8 μM phycocyanin treatment. IKKα and IKKβ served as the catalytic subunits of IκBα, an inhibitory protein of p65. Phosphorylation of IKKα/β could phosphorylate the IκBα protein at Ser32, resulting in the ubiquitin-mediated proteasome-dependent degradation of IκBα, followed by p65 phosphorylation and NF-κB activation [28]. As shown in Figure 7, although the total amounts of IKKα/β, IκBα, and p65 remained stable, their phosphorylation levels (phospho-IKKα/β-Ser176/180, phospho-IκBα-Ser32, and phospho-p65-Ser536) were significantly decreased after phycocyanin treatment, suggesting that the activity of NF-κB signaling was inhibited by phycocyanin. These results demonstrated that phycocyanin could significantly suppress the activity of NF-κB signaling in NSCLC cells. In addition, phycocyanin could also attenuate the phosphorylation levels of AKT in H1299, H460, and LTEP-A2 cells. 

**Figure 8:**
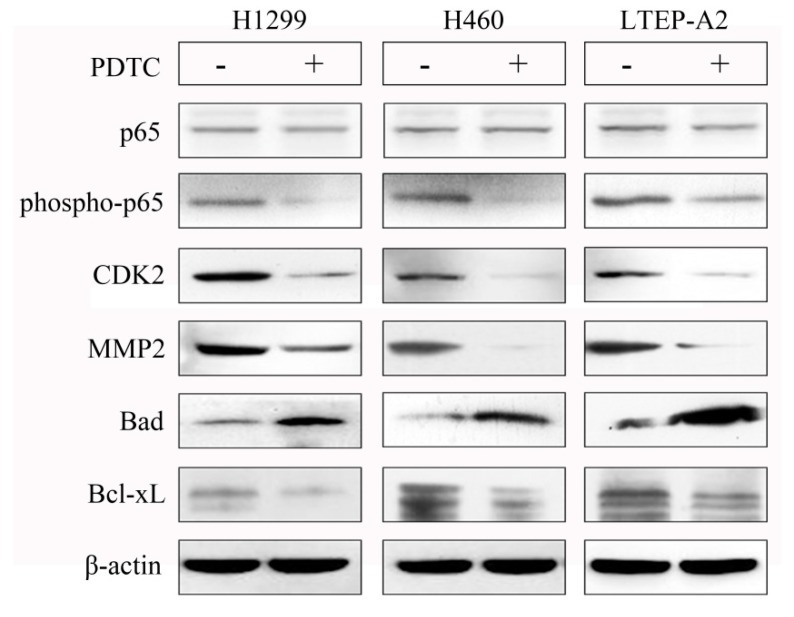
NF-κB signaling has regulatory effect on phenotypic factors of NSCLC cells. The protein expressions of phospho-p65, total p65, CDK2, MMP2, Bad, and Bcl-xL were analyzed by Western blot at 48 h in H1299, H460, and LTEP-A2 cells after 10 μM PDTC treatment for 24 h. PDTC, pyrrolidine dithiocarbamate.

To further explore whether NF-κB was related to phenotypic factors of NSCLC cells, we performed a pyrrolidine dithiocarbamate (PDTC, NK-κB inhibitor) addition experiment. As shown in Figure 8, although the total p65 protein amounts remained stable, the phosphorylation levels of p65 were decreased after 10 μM PDTC treatment, indicating that PDTC inhibited the NF-κB pathway activity of H1299, H460, and LTEP-A2 cells. Strikingly, the protein levels of CDK2, MMP2, and Bcl-xL were downregulated, and the amount of Bad was upregulated after PDTC addition. These results strongly suggested that NF-κB had a regulatory effect on the phenotypic factors of NSCLC cells. Taken together, our study demonstrated that phycocyanin could inhibit the migration and proliferation, as well as promote the apoptosis of H1299, H460, and LTEP-A2 cells through regulating the NF-κB pathway. Although the precise mechanism still needs further investigation, the present research illuminated the function of phycocyanin in NSCLC cells and provided solid evidence that warrants further exploration of the anti-cancer mechanism of phycocyanin.

## 3. Discussion

Over the past few decades, the application of natural products for chemoprevention and therapy has gained great importance [29,30,31]. More and more studies have demonstrated that pharmacological active marine-derived compounds have potent biological activity with little or no side effects [32,33,34]. Phycocyanin, a type of phycobiliprotein derived from *Spirulina*, is one of the compounds that have considerable anti-cancer effects on solid malignancies [35,36]. Non-small cell lung cancer, an extremely aggressive form of cancer with few effective therapeutic options, has attracted that attention of many investigators. Previous studies have suggested that phycocyanin exerts an inhibitory effect on A549 cells, a type of NSCLC cell line [21,22,23]. In this study, we demonstrated that phycocyanin inhibits the growth of H1299, H460, and LTEP-A2 NSCLC cell lines. These results are consistent with previous studies showing that phycocyanin can suppress the proliferation of a variety of tumor cell lines [12,13,37]. To the best of our knowledge, this is the first study to demonstrate the anti-cancer effect of phycocyanin on these NSCLC cell lines, which also highlights the possible mechanism underlying phycocyanin’s cytotoxic and anti-metastatic effects. Our study clearly demonstrated that phycocyanin regulated the NF-κB signaling pathway and also altered the expression of proteins involved in cell cycle and cell survival, by which it mediated growth inhibition and apoptosis. 

Cell cycle regulation plays an important role in cell proliferation, differentiation, and apoptosis. It has been reported that cell cycle regulation dysfunction is closely related to the development of tumors [38]. Peyressatre et al. found that the deregulated activity of cyclin-dependent kinases (CDKs) contributes to altered cellular proliferation in a wide variety of human cancers [39]. What is interesting is that in the present study, phycocyanin was discovered to induce S phase arrest in H1299 and H460 cell lines, while it caused G1 phase arrest in LTEP-A2 cells (Figure 6), which indicated different regulation mechanisms of phycocyanin in different NSCLC cell lines. In fact, phycocyanin could act as an anti-cancer compound through different mechanisms in different types of tumors. It has been reported that phycocyanin induces G1 cell cycle arrest in colon cancer HT-29 cells [7], breast cancer MDA-MB-231 cells [6], and chronic myelocytic leukemia K562 cells [40]. Meanwhile, it could also block G2/M cell cycle progression in pancreatic cancer PANC-1 cells [41], ovarian cancer SKOV-3 cells [14], and liver cancer HepG2 cells [42]. Thangam et al. reported that phycocyanin could induce G1 phase arrest in lung adenocarcinoma A549 cells [7], which is in agreement with the result of LTEP-A2 cells in our study. It is worth noting that H1299 and LTEP-A2 both belong to lung adenocarcinoma cell lines, but the cell cycles are restrained in different phases. Interestingly, Yao et al. discovered that pseudolaric acid B, a diterpene acid isolated from the root of *Pseudolarix kaempferi*, could inhibit the growth and cause G2/M arrest of A549 cells, but has no effect on H1299 cells because H1299 is a type of p53 null cell line [43]. Therefore, we speculate that p53 might be involved in phycocyanin-induced cell cycle arrest in H1299 (p53 null) and LTEP-A2 (p53 wild type) cells. However, the underlying mechanism still needs further investigation. Taken together, our study provided useful information on the regulation approach of phycocyanin in NSCLC cells. 

Cells arrested in mitosis upon the activation of mitotic catastrophe have different fates, including death during mitosis or the entrance into the subsequent cell cycle followed by cell death [44], indicating that cell cycle regulation is closely related to apoptosis. In the present study, phycocyanin was found to induce apoptosis in NSCLC cells. The apoptotic percentage of H460 cells reached 3.72 ± 0.98% with phycocyanin treatment, which was significantly different than that in control cells, but markedly lower than that in H1299 (11.3 ± 0.16%) and LTEP-A2 cells (14.5 ± 0.68%). It is worth noting that Shin et al. discovered that (E)-2-benzylidene-3-(cyclohexylamino)-2,3-dihydro-1H-inden-1-one (BCI, an inhibitor of dual specific phosphatase 1/6 and mitogen-activated protein kinase) significantly inhibits the viability of H1299 cells as compared to H460 cells [45], suggesting that different mechanisms might exist in lung adenocarcinoma (H1299 and LTEP-A2 cells) and undifferentiated large cell lung carcinoma (H460 cells) cell lines. Particularly, the transcription and protein levels of two apoptotic markers (Bcl-2 and Bcl-xL) were not consistently expressed in H460 cells (Figure 3), which further supports the above hypothesis. Interestingly, although the degree of apoptosis in H460 cells is low, it shows the highest expression level of p21 (Figure 6B). It is known that the growth inhibition and the expression of regulation factors might show discrepancy in some cases. Tsui et al. investigated the anti-cancer functions of flavonoids in H460 and A549 cells, and discovered that although H460 cells are more susceptible to flavonoids than A549, p53 level was constitutive and not significantly altered [46]. In the present study, despite the fact that the apoptotic proportion of H460 was low at 48 h after phycocyanin treatment, the proliferation rate dramatically decreased after the third day (Figure 5B). In this case, we speculate that the increased p21 might cause a delay in cell growth and activity inhibition, in spite of the low apoptotic ratio at 48 h. 

NF-κB signaling, one of the classical pathways in cells, has been reported to regulate proliferation and apoptosis in various kinds of cancers [47]. Recent studies have demonstrated that the NF-κB pathway is involved in phycocyanin-induced growth inhibition in liver and pancreatic carcinoma cells [41,48]. However, few researchers reported the relationship between NF-κB and phycocyanin in lung cancer; only Bingula et al. discovered that the combined treatment of phycocyanin and betaine could reduce the stimulation of NF-κB expression in A549 cells [23]. In the present study, phycocyanin was first discovered to reduce the activity of NF-κB signaling in H1299, H460, and LTEP-A2 cells (Figure 7), which suggests that phycocyanin could suppress proliferation and induce apoptosis through the inactivation of the NF-κB pathway in NSCLC cell lines. 

Cancer metastasis is a complex and multistep event, wherein cancer cells leave the site of the primary tumor and disseminate to distant sites in the body [49]. It is the most destructive stage of cancer progression and the leading cause of cancer-related deaths [50]. More recently, extensive attention has been drawn toward phycocyanin for its potent migration inhibition effect on cancer cells. It has been reported that phycocyanin could suppress the migration of breast cancer and melanoma cells through MAPK signaling [6,51]. Our study showed that phycocyanin exerted a remarkable migration inhibition effect on different NSCLC cells through regulating MMP2 and MMP9 for the first time (Figure 4). Strikingly, NF-κB is reported to play a key role in tumor migration [52] and has a synergistic expression pattern with MMP2 [53], which suggests that MMP2/NF-κB could be involved in phycocyanin-induced migration inhibition regulation in NSCLC cells. 

## 4. Materials and Methods

### 4.1. Materials and Reagents

Dulbecco’s modified Eagle’s medium (DMEM) was purchased from Invitrogen (Carlsbad, CA, USA). Phycocyanin (derived from *Spirulina platensis*) standard substance was purchased from Envirologix (Portland, ME, USA). Fetal bovine serum was purchased from Hyclone (Logan, UT, USA). The cell culture consumables were purchased from Corning (Tewksbury, MA, USA). Cell apoptosis analysis kit, RIPA lysis buffer, and protease and phosphatase inhibitors were purchased from Roche (Mannheim, Germany). Propidium iodide, RNase, and skim milk were purchased from Becton Dickinson (Franklin Lakes, NJ, USA). Polyvinylidene difluoride (PVDF) membrane and enhanced chemiluminescence (ECL) kit were purchased from Millipore (Schwalbach, Germany). Antibodies were purchased from Cell Signaling Technology (Danvers, MA, USA). PrimeScript RT Master Mix was purchased from Takara (Dalian, China). 

### 4.2. Cell Line and Culture Conditions

Human NSCLC cell lines NCI-H1299, NCI-H460, and LTEP-A2 were purchased from American Type Cell Collection (ATCC, Manassas, VA, USA). All cell lines were cultured in DMEM media supplemented with 10% heat-inactivated fetal bovine serum (FBS), 0.1 mg/mL of streptomycin, and 100 units/mL of penicillin at 37 °C in a humidified atmosphere with 5% CO_2_. Cells were sub-cultured every 3–5 days. Cells between 3–15 passages were used in all experiments.

### 4.3. Cell Viability Assay

The cell viability was performed by MTT assay. Briefly, cells were seeded at a density of 5000 cells in 100 μL of complete medium per well into 96-well plates. After 12 h of incubation for cell attachment, phycocyanin was added to each well with a final concentration of 0, 1.2, 2.4, or 4.8 μM. Control cells were treated with equal volumes of phosphate buffer solution (PBS). After incubation for 24 h, the culture medium was supplemented with 1 mg/mL MTT for 4 h at 37 °C. The medium was removed and the cells were solubilized with DMSO. The absorbance was then measured at 450 nm and 630 nm. The results were expressed as a survival rate of the absorbance reading of the control cells.

### 4.4. Cell Proliferation Assay

Cell proliferation was determined by MTT assay. Briefly, cells were seeded at 5000 cells in 100 μL of complete medium per well in quadruplicate in 96-well plates. After 12 h of incubation for cell attachment, phycocyanin was added to each well with a final concentration of 4.8 μM. Each day, 10 μL MTT/well was added to test cells and incubated for 4 h at 37 °C. Then SDS-HCl solution (10% SDS, 0.01 M HCl) was added into each well and incubated for 14 h at 37 °C. The absorbance of formazan was measured at a wavelength of 570 nm using a microplate reader. The assay lasted for 5 or 6 days after treatment. Three independent experiments were performed.

### 4.5. Colony Formation Assay

Cells in the exponential growth phase were harvested and seeded at about 300 cells per well in six-well plates. After 12 h incubation, cells were treated for another 24 h with 0, 2.4, or 4.8 μM phycocyanin, and then continuously incubated in fresh medium at 37 °C in 5% humidified CO_2_. After incubation for 10–14 days, cells were washed with PBS twice, fixed with methanol for 15 min, and stained with 0.5% crystal violet for 15 min at room temperature. The number of colonies was counted for analysis.

### 4.6. Cell Apoptosis Assay

After being treated with phycocyanin (0, 2.4, and 4.8 μM) for 48 h, cells were harvested and washed twice with cold PBS and then resuspended gently in 500 μL binding buffer. Thereafter, cells were stained in 5 μL Annexin V-FITC/7-aminoactinomycin D (7-AAD) according to the manufacturer’s protocol. Stained cells were analyzed by FACSCalibur (Becton Dickinson).

### 4.7. Cell Cycle Assay

After treatment with phycocyanin for 48 h, cells were harvested and fixed in 1 mL 70% cold ethanol in test tubes and incubated at 4 °C for at least 48 h. Cells were centrifuged at 1500 rpm for 5 min and the cell pellets were resuspended in 500 μL of PI/RNase staining buffer, incubated on ice for 30 min, and washed twice with cold PBS. Cell cycle distribution was measured using FACSCalibur (Becton Dickinson).

### 4.8. Western Blot Analysis

Proteins were extracted by RIPA lysis buffer (1% NP40, 0.1% SDS, 5 mM EDTA, 0.5% sodium deoxycholate, 1 mM sodium orthovanadate) containing protease and phosphatase inhibitors. Equivalent amounts of proteins were separated by 12% SDS-PAGE, and then electro-transferred onto PVDF membranes. After blocking with 5% skim milk, the membranes were incubated with primary antibodies at 4 °C overnight, followed by incubation with horseradish peroxidase-conjugated secondary antibodies. Signals were detected by an ECL system.

### 4.9. Quantitative RT-PCR

Total RNA was extracted using Trizol reagent and reverse-transcribed with PrimeScript RT Master Mix. Real-time PCR analysis was performed in an Applied Biosystems Step One-Plus (Waltham, MA, USA) under the following conditions: 95 °C for 30 s, followed by 40 cycles at 95 °C for 5 s, and 60 °C for 40 s. The relative expression of each targeted gene was calculated and normalized using 2^−ΔΔCt^ method relative to glyceraldehyde-3-phosphate dehydrogenase (GAPDH). Each assay was performed in quadruplicate.

### 4.10. Wound-Healing Assay

Cells in the exponential growth phase were harvested and seeded in six-well plates. After 12 h incubation, cells were treated for another 24 h with 0, 2.4, or 4.8 μM phycocyanin, and then continuously incubated in fresh medium (containing 3% fetal bovine serum) at 37 °C in 5% humidified CO_2_. The culture insert provided two cell culture reservoirs that were separated by a thick wall. After removing the culture inserts on the second day, a “wound” was formed between the two cell patches. Photos of the wounds were taken every 12 h. The widths of the wounds were measured at three positions for each replicate using the Leica Application Suite (Leica Microsystems GmbH, Wetzlar, Germany). 

### 4.11. Statistical Analysis

The numerical data were expressed as means ± standard deviation (SD). Two-tailed Student’s *t*-test was performed for comparison among the different groups. In addition, *p* < 0.05 (*) or *p* < 0.01 (**) was considered as statistically significant. 

## 5. Conclusions

The present study first demonstrated that phycocyanin, a natural functional food colorant, inhibited migration, proliferation, and promoted apoptosis in three NSCLC cell lines (H1299, H460, and LTEP-A2). Molecular studies further revealed that NF-κB was involved in this process. Phycocyanin could affect the cell phenotype through downregulating the NF-κB pathway. Although the precise mechanism still needs further investigation, the present findings illuminated the function of phycocyanin in NSCLC cells and provided solid evidence to warrant the further exploration of the anti-cancer mechanism of phycocyanin.
